# Research on Urban Medical and Health Services Efficiency and Its Spatial Correlation in China: Based on Panel Data of 13 Cities in Jiangsu Province

**DOI:** 10.3390/healthcare9091167

**Published:** 2021-09-06

**Authors:** Lingling Lin, Fang Wu, Wei Chen, Chenming Zhu, Tao Huang

**Affiliations:** School of International Pharmaceutical Business, China Pharmaceutical University, Nanjing 211198, China; L531568809@163.com (L.L.); cw2017_cpu@163.com (W.C.); zcm18795993365@163.com (C.Z.); ht15298360992@126.com (T.H.)

**Keywords:** medical and health services, efficiency, data envelopment analysis, exploratory spatial data analysis, spatial autocorrelation

## Abstract

The improvement of the efficiency of medical and health services is of great significance for improving the high-quality and efficient medical and health services system and meeting the increasingly diverse health needs of residents. Based on the panel data of 13 cities in Jiangsu Province, this research analyzed the relative effectiveness of medical and health services from 2015 to 2019 using the super efficiency slack-based measure-data envelopment analysis model, and the Malmquist index method was used to explore the changes in the efficiency of medical and health services from a dynamic perspective. Furthermore, the spatial autocorrelation analysis method was used to verify the spatial correlation of medical and health services efficiency. In general, there is room for improvement in the efficiency of medical and health services in 13 cities in Jiangsu Province. There are obvious differences in regional efficiency, and there is a certain spatial correlation. In the future, the medical and health services efficiency of China’s cities should be improved by increasing the investment in high-quality medical and health resources, optimizing their layout and making full use of the spatial spillover effects between neighboring cities to strengthen inter-regional cooperation and exchanges.

## 1. Introduction

Efficient and equitable delivery of services is an important goal of health systems [1]. The efficiency of medical and health services is an important issue related to the people’s sense of contentment, well-being, and security. The core is how to rationally allocate the limited medical and health resources invested so as to maximize the effective output of medical and health services that can meet the diverse needs of residents [2,3]. Since the new health-care reform, China’s investment in medical and health services has increased steadily. The total national health expenditure increased from 1754.192 billion yuan in 2009 to 6584.139 billion yuan in 2019, with an annual growth rate of 11.37% to 21.85%. The proportion of total health expenditure in GDP has also increased year by year, from 5.03% in 2009 to 6.64% in 2019 [4]. Under the great attention of the state, the scale of China’s medical and health resources investment has increased significantly. The data from National Bureau of Statistics of the People’s Republic of China indicate that the number of medical and health institutions increased from 916,571 in 2009 to 1,007,579 in 2019, and the number of health personnel increased from 778,148 in 2009 to 12,928,335 in 2019, an increase of 66.14%. The medical and health services network covering both urban and rural areas has been gradually improved and the people’s health condition has continued to improve. However, at the same time, with the gradual improvement of the medical security system, the acceleration of industrialization and urbanization and the increasing aging problem, the demand for residents’ medical and health services has been released to a greater extent, which has led to a gap between it and the limited supply of medical and health resources. The contradiction continues to worsen. At present, there are still some problems, such as unbalanced development of medical and health services between urban and rural areas, unequal access to medical and health resources between regions, and mismatch between low health output and high health input [5,6]. On 13 March 2021, the Outline of the 14 Five-Year Plan (2021–2025) for National Economic and Social Development and the Long-Range Objectives Through the Year 2035 of the People’s Republic of China came out, which emphasizes the need to improve the quality and efficiency of medical care, expand the supply of medical service resources, and speed up the expansion of high-quality medical resources and the balanced distribution of regions [7]. However, considering the scarcity of high-quality medical and health resources and the substantial increase in residents’ demand for medical and health services, how Chinese cities can further rationally allocate medical and health resources and improve the efficiency of medical and health services during the “14th Five-Year Plan” period became an urgent problem to be solved.

Scientifically and accurately measuring the efficiency of medical and health services is an evidence-based precondition for discovering and solving problems of Chinese cities. In terms of efficiency measurement, commonly used methods include non-parametric methods such as Data Envelopment Analysis (DEA) and parametric methods such as Stochastic Frontier Analysis (SFA), the latter of which is used less frequently than the former [8,9]. Among them, most focus on analyzing the efficiency of hospitals and healthcare centers [10]. Compared with the stochastic frontier analysis method, the data envelopment analysis method is a widely used non-parametric method. The main advantage is that it does not need to make assumptions about the form of the function, and it can contain multiple inputs and outputs measured in different units simultaneously [11,12]. Of course, this is also one of the main considerations in choosing the DEA method in this study. Since Sherman [13] first applied the DEA method to the medical and health field to evaluate the efficiency of medical and health care, many scholars have used a more mature DEA method to carry out studies related to efficiency evaluation. For example, Campos et al. [14] used the DEA model to evaluate the efficiency of the Spanish autonomous community health systems. Ozcan et al. [15] used a dynamic network DEA model to analyze the efficiency of public health system and medical care system in OECD countries. Mohamadi et al. [16] used the Malmquist index model to evaluate and analyze the performance of the Iranian health system’s efficiency of using health resources. Giménez et al. [17] analyzed the efficiency of Colombian health system hospitals by calculating the Directional Distance Function and the global Mamlquist–Luenberger Index from both static and dynamic perspectives. Tigga et al. [18] used traditional DEA methods to measure and compare the efficiency of medical systems in 27 Indian states regarding states as decision-making units. Chinese scholars have also achieved certain results in research projects on efficiency of medical and health services. In terms of research objects, scholars have carried out research on the efficiency of medical and health services in different administrative divisions such as provinces, districts, and cities [19,20,21]. When selecting specific models, most scholars tended to use traditional DEA models, such as the Banker, Charnes, and Cooper (BCC) model, as well as the Charnes, Cooper, and Rhodes (CCR) model, to evaluate and analyze the efficiency during a certain period of time [22,23,24]. However, the accuracy of the efficiency value measured by the traditional DEA model is low. There is a certain degree of underestimation, and it cannot reflect the inter-period change trend of efficiency [25]. Therefore, some scholars used the multi-stage DEA model, slack-based measure (SBM) model, and super-efficiency DEA model to evaluate and analyze efficiency [26,27,28]. Some scholars may also choose dynamic models such as DtSBM model and Malmquist index for cross-period efficiency evaluation and analysis [29,30].

On the whole, the existing literature about the evaluation and analysis of medical and health services efficiency is relatively abundant, which has laid an important foundation for a related study. However, previous studies mostly focused on the evaluation and analysis of efficiency. The analysis of the spatial correlation of medical and health services efficiency was relatively lacking. Therefore, this research took Jiangsu Province as an example, combined panel data from 2015 to 2019, and used urban medical and health resources as input, and medical and health services as output. Firstly, the super efficiency slack-based measure model (SE-SBM) model and the Malmquist index method were used to measure the efficiency of medical and health services in an all-round way, comprehensively reflecting the overall efficiency level, regional differences, and deep-seated influencing factors from both static and dynamic inclusive perspective. Then, the exploratory spatial data analysis (ESDA) method was applied to analyze the spatial autocorrelation of efficiency and deeply explore the spatial correlation and agglomeration characteristics of efficiency. In addition, with the help of ArcGIS software, the study results could be visualized and relevant data could be displayed clearly and intuitively. The main objective of this research was to provide reference for Chinese cities to improve the efficiency of medical and health services and promote the coordinated development of regional medical and health undertakings.

The remainder of this study is organized as follows. Section 2 illustrates the study area, measurement methods, indicators, and data sources. Section 3 presents the empirical results of the comprehensive evaluation of medical and health services efficiency, including the results of the DEA-SE-SBM model and the DEA-Malmquist model. The results of spatial correlation analysis of the efficiency of medical and health services are presented in Section 4. Section 5 presents the discussion. The conclusions are presented in in Section 6.

## 2. Materials and Methods

### 2.1. Study Areas

**Figure 1:**
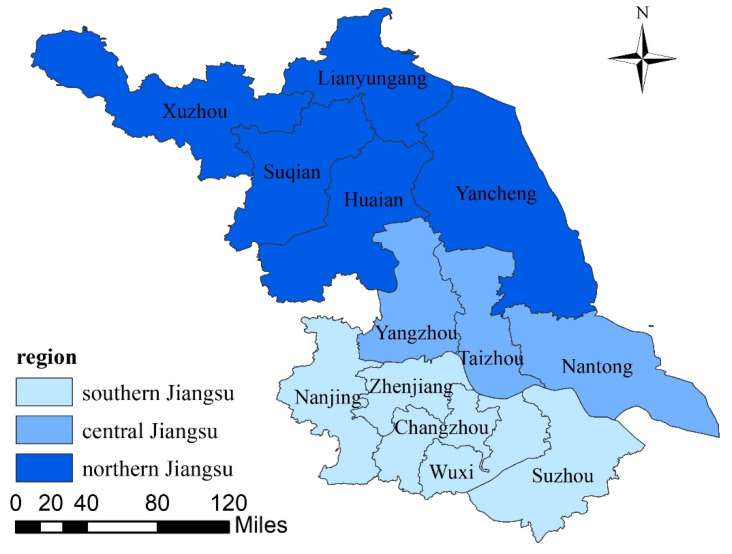
Distribution map of southern, central, and northern regions in Jiangsu Province.

In 2015, China’s national level clearly defined the reform of the medical and health system at the provincial level for the first time, and Jiangsu Province was one of the first batches of pilot provinces for comprehensive medical reform. It is located in the lower reaches of the Yangtze River and has 13 prefecture-level cities (shown in Figure 1 and Table 1). Among them, 9 prefecture-level cities belong to the Yangtze River Delta City Group. According to spatial geography and the classification in the official website of the People’s Government and Jiangsu Health Yearbook, the province can be divided into three regions: southern Jiangsu, central Jiangsu, and northern Jiangsu. As China’s second largest economy (Jiangsu Province’s GDP in 2019 was 9963.151 billion yuan, second only to Guangdong (10,767.107 billion yuan)) and one of the 11 comprehensive medical reform pilot provinces, Jiangsu Province has a relatively rich stock of medical and health resources. By the end of 2019, the number of beds per thousand population had reached 6.39 and the number of health technical personnel per thousand population had reached 7.85, ranking among the top in the country. However, the allocation of medical and health resources in the province was uneven. The number of beds per thousand people in southern Jiangsu, central Jiangsu, and northern Jiangsu were 6.67, 6.14, and 6.22, respectively, and the number of health technical personnel per thousand people were 8.97, 6.75, and 7.20. The level of southern Jiangsu was significantly higher than the average while the level of the northern Jiangsu was lower. The efficiency of medical and health resource allocation had obvious differences and regional characteristics [21]. Therefore, this study takes Jiangsu Province as an example of empirical objects, comprehensively measures the efficiency of medical and health services in 13 cities in the province during the five years since 2015, and deeply analyzes the global and local spatial differences and relevance of efficiency. It aims to provide an important reference for formulating and implementing policies related to improving the quality and efficiency of urban medical and health services, and making contributions to improve the accessibility and efficiency of residents’ medical and health services.

### 2.2. Methods

#### 2.2.1. Data Envelopment Analysis

(1) DEA-SE-SBM Model

DEA is a more mature method among non-parametric efficiency analysis methods. Its traditional models include CCR, BCC, and other models [31,32], which have the following two shortcomings: one is that they are all measured based on a radial angle, without considering the slack variables of input and output elements [33]. In the medical and health service systems, the outputs, such as the number of discharged patients and the utilization rates of beds, do not change proportionally [27]. Therefore, the measurement results may not be accurate enough. The second is that the measured efficiency value ranges from 0~1; units that simultaneously achieve efficiency (efficiency value is 1) cannot be compared [34]. Based on the traditional DEA model, Tone K proposed a non-radial, non-angle slack-based measure model (SBM), which can effectively compensate for the defect that some traditional models do not include slack variables in the measurement of inefficiency and improve accuracy [35]. Furthermore, Tone K then combined the SBM model with the super efficiency model and proposed the super efficiency slack-based measure model (SE-SBM), which can further compare decision-making units (DMUs) that achieve efficiency value of 1 at the same time, so as to make up for the second shortcoming mentioned above [36]. Therefore, this study adopted the DEA-SE-SBM model based on the measurement of input slack with the intention of accurately reflecting the medical and health service efficiency of the 13 prefecture-level cities in Jiangsu Province. The formula is shown in (1).

(1) $$\begin{array}{l} \rho^{*}=\min\rho =\frac{1-\left(1/m\right)\sum_{i=1}^{m}\frac{s_{i}^{-}}{x_{ik}}}{1+\left(1/z\right)\sum_{r=1}^{z}\frac{s_{r}^{+}}{y_{rk}}}\\ s.t.\left\{ \begin{array}{l} x_{ik}=\sum_{j=1,j\neq k}^{n}x_{ij}\lambda_{j}+s_{i}^{-}\\ y_{rk}=\sum_{j=1,j\neq k}^{n}y_{rj}\lambda_{j}-s_{r}^{+}\\ \lambda_{j},s_{i}^{-},s_{r}^{+}>0\\ i=1,2,\cdots ,m\\ r=1,2,\cdots ,z\\ j=1,2,\cdots ,n;j\neq k \end{array}\right. \end{array}$$

In this formula, *n* is the number of measured cities (*n* = 13 in this study), *j* represents the *j*-th city (*j* = 1, 2…*n*), each city has *m* inputs and *z* outputs. *x_ij_* and *y_r_*_j_ represent the *i*-th input and the *r*-th output of the *j*-th city. *x_ik_* and *y_rk_* represent the *i*-th input and the *r*-th output of the *k*-th city. ρ^*^ represents the efficiency value of each city. *λ* represents the weight vector; $s_{i}^{-}$ and $s_{i}^{+}$ are slack variables of the input and output vectors, which respectively represent the amount of savings in medical and health resources input and the amount of increase in medical and health services output in tested cities.

Furthermore, according to model (1), it is possible to calculate the medical and health service efficiency of 13 cities in Jiangsu Province from 2015 to 2019. As shown in Formula (2), *Score_kt_* represents the medical and health services efficiency of city *k* in year *t*.

(2) $$Score_{kt}=\rho_{kt}^{*}(k=1,2,\cdots 13;t=2015,\cdots 2019)$$

(2) DEA-Malmquist Model

In terms of efficiency evaluation, the DEA-SE-SBM model can only measure the efficiency in a certain period of time. It cannot continuously compare different time sections so that it is unable to reflect the dynamic evolution characteristics of efficiency. Therefore, this study used the DEA-Malmquist model to further calculate the Total Factor Productivity Change (TFPCH) of medical and health services to reveal the dynamic evolution. Let (*x^t^*, *y^t^*) and (*x^t+1^*, *y^t+1^*) denote the input-output vector of the medical system in period *t* and *t+1*, respectively, then the TFPCH of the two adjacent periods is:

(3) TFPCH=M(xt,yt,xt+1,yt+1)=[Dt(xt+1,yt+1)Dt(xt,yt)×Dt+1(xt+1,yt+1)Dt+1(xt,yt)]12

In this formula, *D^t^* (*x^t^*, *y^t^*) and *D^t^* (*x^t+1^*, *y^t+1^*), respectively, represent the distance function of the expected city in period *t* and *t+1* when period *t* is used as the technical reference. *D^t+1^*(*x^t^*, *y^t^*) and *D^t+1^*(*x^t+1^*, *y^t+1^*) have similar meanings. According to the decomposition of Malmquist index by Fare et al. [37], it can be further decomposed into two parts: Technical (Comprehensive) Efficiency Change index (EFFCH) and Technical Change index (TECHCH). Furthermore, with variable returns to scale (VRS) as the hypothesis, EFFCH can be decomposed into Pure Efficiency Change index (PECH) and Scale Efficiency Change index (SECH). If *TEPCH >* 1, it means that the total factor production efficiency of the tested object has increased during period *t* to *t +* 1. If *TEPCH <* 1, it means a decline. If *TEPCH =* 1, it means it remains unchanged. *TECHCH >* 1 represents technical progress and vice versa. PECH represents the change in the management level that causes the efficiency to change, and *PECH >* 1 represents the improvement of the management level and vice versa. *SECH >* 1 represents the optimization of scale efficiency and vice versa.

#### 2.2.2. Exploratory Spatial Data Analysis

ESDA is a commonly used research method in spatial econometrics, which is mainly used to study the correlation between a phenomenon and the attribute values of its neighboring units in geographic space [38]. The spatial autocorrelation analysis in ESDA is the analysis of the potential interdependence and correlation degree between certain observations within a certain range. It can measure the aggregation or dispersion of the spatial element attributes of the study object [39]. It mainly includes global spatial autocorrelation analysis and local spatial autocorrelation analysis [40]. This study used global and local spatial autocorrelation analysis to reveal the overall spatial correlation and internal correlation characteristics of the medical and health services efficiency in a comprehensive manner.

(1) Global Spatial Autocorrelation

Global spatial autocorrelation analysis is used to describe the spatial correlation and difference degree of variable observations in the entire study area. This study used Geoda 1.16 software to calculate the global Moran’s *I* to quantify the overall spatial correlation degree of the medical and health services efficiency. The calculation formula is as follows:

(4) $$I=\frac{n\sum_{j=1}^{n}\sum_{k=1}^{n}W_{jk}\left(\rho_{j}^{*}-\bar{\rho^{*}}\right)\left(\rho_{k}^{*}-\bar{\rho^{*}}\right)}{\sum_{j=1}^{n}\sum_{k=1}^{n}W_{jk}\sum_{j=1}^{n}{\left(\rho_{j}^{*}-\bar{\rho^{*}}\right)}^{2}}$$

In this formula, *I* is the global Moran’s *I*; $\rho_{j}^{*}$ and $\rho_{k}^{*}$ are the medical and health services efficiency values of prefecture-level city *j* and *k*. *W_jk_* is the spatial weight matrix, which used to measure the spatial relationship between prefecture-level city *j* and *k*; this study uses the Rook spatial adjacency. $\bar{\rho^{*}}$ is the average value of medical and health services efficiency. The value range of global Moran’s *I* is [−1,1]. When *I* > 0, it means that there is a positive spatial correlation in efficiency; that is to say, cities with higher (or lower) medical and health services in the province tend to be spatially more concentrated. When *I* < 0, it means that there is a negative spatial correlation. That is, there is a spatial difference in the efficiency of medical and health services between each city and its surrounding cities. When *I* = 0, it indicates that there is no spatial correlation in efficiency; that is, the efficiency of each city’s health services presents a random distribution in space [41]. In addition, a standardized *Z*-statistic test method is needed to determine whether the global Moran’s *I* is significant.

(2) Local Spatial Autocorrelation

Local spatial autocorrelation analysis is used to analyze the degree of spatial correlation between each spatial object and its neighboring units in certain areas to reflect the local feature differences in the distribution of spatial objects. It can effectively compensate for the global spatial autocorrelation analysis using only a single value to reflect the degree of spatial association of the study object and then ignore the potential instability defects in the local space [42]. This study will calculate local Moran’s *I* to reveal the degree of correlation of the medical and health services efficiency between a certain city and its neighboring cities. For a prefecture-level city *j*, the local Moran’s *I* index can be expressed as:

(5) $$I_{j}=\frac{n\left(\rho_{j}^{*}-\bar{\rho^{*}}\right)}{\sum_{j=1}^{n}{\left(\rho_{j}^{*}-\bar{\rho^{*}}\right)}^{2}}\sum_{k=1}^{n}W_{jk}\left(\rho_{k}^{*}-\bar{\rho^{*}}\right)$$

The variables involved in the above formula have the same meaning as those in Formula (4). When *I_j_* is a positive value, it means that the attributes of the city and neighboring cities are similar, and when *I_j_* is a negative value, it means that they are not similar. At the same time, this study will visualize the calculation results by drawing a Moran scatter plot and a local indicator of spatial association (LISA) agglomeration map. The Moran scatter plot uses the standardized variable (*z*) of the efficiency value as the horizontal axis and the spatial lag variable (*W_z_*) as the vertical axis to describe the local correlation of the research object in the form of a scatter plot. The spatial correlation model of medical and health services efficiency can be divided into four categories by the four quadrants of the scatter plot. The first and third quadrants represent the spatial agglomeration effect. The first quadrant is the agglomeration area, which is used to represent the relationship that high-efficiency cities are surrounded by high-efficiency cities (H-H agglomeration). The third quadrant is the depression area, indicating that low-efficiency value cities are surrounded by low-efficiency cities (L-L agglomeration). The second and fourth quadrants represent spatial differentiation effects. The second quadrant is a hollow area, which is used to indicate the relationship that low-efficiency cities are surrounded by high-efficiency cities (L-H agglomeration). The fourth quadrant is an island area, which represents the relationship that high-efficiency cities are surrounded by low-efficiency cities (H-L agglomeration) [43]. The LISA agglomeration map visually shows the type of local spatial autocorrelation between each city and its neighboring cities [44].

### 2.3. Selection of Indicators and Data Sources

In terms of investment indicators, medical and health resources can be roughly divided into three categories: financial resources, human resources, and material resources [45]. Among them, financial resources are the government’s financial investment in the field of medical and health services; its direct output increases human and material resources of the medical and health, which belong to the first stage of input and output in this field. The input and output of the second stage is the input of human and material resources, which in turn affects the changes in the amount of medical and health services. This study evaluated the efficiency of the second stage, that is, the efficiency of medical and health services, so the relevant elements of human and material resources of the medical and health were selected as input indicators. Existing studies usually use the number of health personnel, the number of health technical personnel, the number of registered nurses, and the number of licensed (assistant) physicians to represent human resource input, and use the number of beds, the number of health institutions, and the number of equipment above 10,000 yuan to represent material resource input [29,46,47]. In terms of output indicators, due to the complexity and particularity of the medical and health industry, its output is usually measured by disease cure and improvement of people’s health, but these are difficult to quantify. In relative studies, outpatient and emergency visits, rates of utilization of beds, and the number of admitted (discharged) patients are used as quantitative indicators of the supply of medical services to reflect the output of medical and health resources [27,28,48].

Based on the above analysis, taking into account the scientific, reliable, and comprehensive principles of the index system construction, the specific situation of Jiangsu Province, and the availability of relevant data, this study selected the number of health technicians, the number of health institutions, and the number of beds as input indicators. The number of outpatients and emergency visits and the utilization rate of beds were selected as output indicators (Table 2).

The data of 2015–2019 in this study come from the *Year Book of Public Health and Family Planning in Jiangsu (2016–2018)* and *Jiangsu Health Yearbook (2019–2020)*. For individual missing data, this study used linear Interpolation to make a supplement. Other data come from the *Statistical Bulletin on the Development of Health Care in Jiangsu in 2019*, *China Health Statistical Yearbook 2020*, and *China Statistical Yearbook (2020)*.

### 2.4. Medical and Health Input-Output in Jiangsu Province

From 2015 to 2019, the medical and health investment in Jiangsu Province showed an overall increase (show in Table 3). In terms of the number of health technicians, as China continues to strengthen the construction of medical and health personnel, cities in Jiangsu Province actively respond to the call; the number of health technicians has a large increase, the largest is Nanjing, which increased from 65,139 in 2015 to 93,856 in 2019, by 44.09%. In terms of the number of health institutions, Xuzhou, Huaian, and Suqian saw negative growth, while other cities saw positive growth. In terms of the number of beds, Taizhou has a larger increase (36.85%), while Zhenjiang has a smaller increase (8.25%). In terms of medical and health services output, the number of outpatient and emergency visits in Huaian and Yangzhou decreased to a certain extent, with a growth rate of −1.60% and −0.15%, respectively. In terms of the utilization rate of beds, most cities showed a decreasing characteristic, among which Xuzhou had a larger decrease (−12.58%). In general, most cities in Jiangsu Province have continuously increased investment in medical and health resources, which is conducive to improving the accessibility of medical and health services. However, the output of medical and health services in some cities has not been increased correspondingly, and the utilization efficiency of medical and health resources needs to be further studied.

## 3. Results of the Comprehensive Evaluation of the Efficiency of Medical and Health Services

### 3.1. Results of the DEA-SE-SBM Model

**Figure 2:**
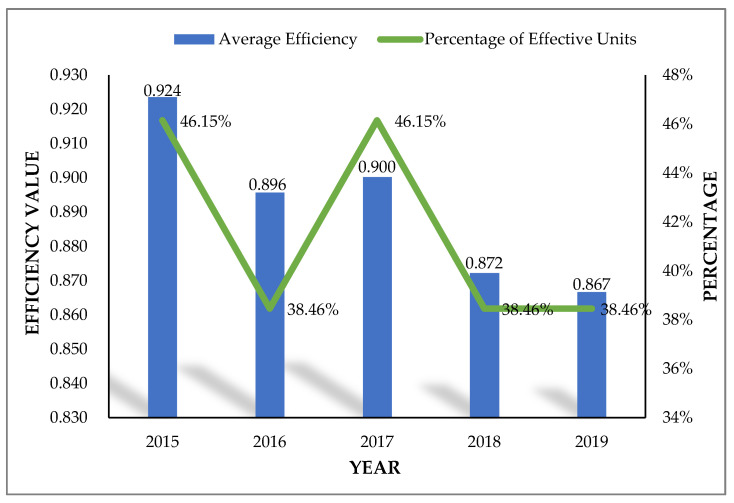
The average efficiency and the percentage of effective units in 13 cities in Jiangsu Province from 2015 to 2019.

On the whole (Table 4), the total average efficiency of medical and health services in Jiangsu Province from 2015 to 2019 was less than 1, ranging from 0.860 to 0.930. It indicates that there is room for improvement in the overall efficiency of medical and health services in this province. From the perspective of time, the province’s average efficiency over the five-year period showed the characteristics of “decline-rise-decline”. To be specific, from 2015 to 2016, the overall average efficiency showed a downward trend (falling from 0.924 to 0.896) and then rose to 0.900 in 2017. However, it successively declined in 2018 and 2019 (0.872 and 0.867). From the perspective of the percentage of effective units per year (Figure 2), the percentage of effective units of 13 cities in Jiangsu Province accounted for 38.46–46.15% from 2015 to 2019; that is to say, the number of cities with effective medical and health services did not exceed half during the study period. It further explains that, in order to improve overall efficiency, most cities in Jiangsu Province need urgent improvement in the allocation of medical and health resources and the supply of medical and health services.

**Figure 3:**
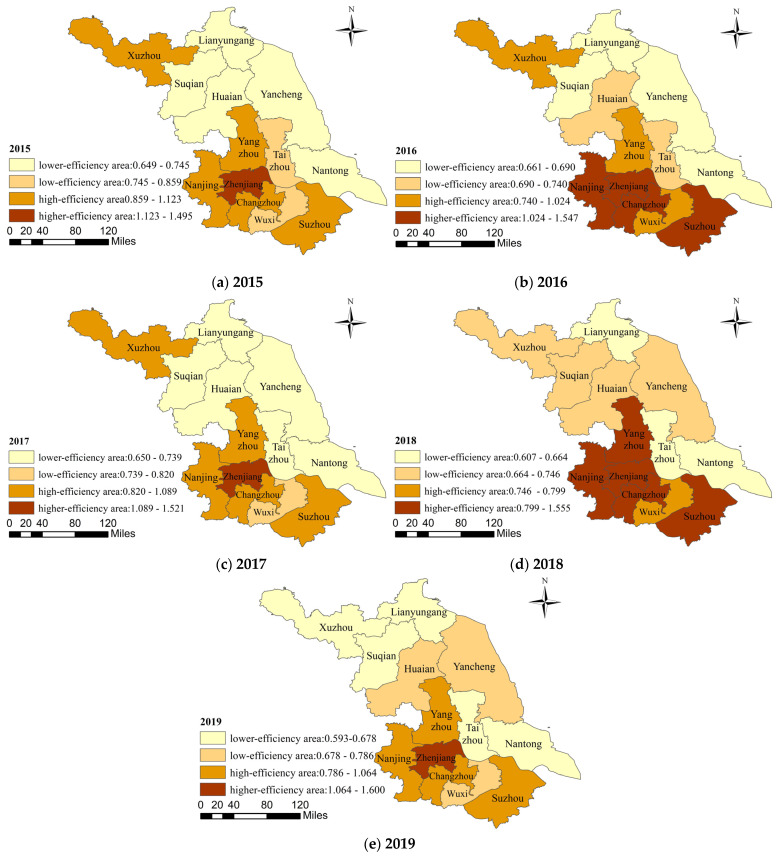
The spatial distribution of medical and health services efficiency in Jiangsu Province from 2015 to 2019.

In order to reflect the spatial distribution characteristics of the medical and health services efficiency in Jiangsu Province more clearly and intuitively, based on the efficiency values of each city from 2015 to 2019, we used the Natural Breakpoint Classification Method in ArcGIS 10.2 software to classify 13 Jiangsu Province into four categories: lower-efficiency area, low-efficiency area, high-efficiency area, and higher-efficiency area (Figure 3).

From a macro perspective, the spatial distribution pattern of medical and health services efficiency in Jiangsu Province was relatively stable during the study period, showing a characteristic of attenuation from south to north and can be concluded to have the trend of “southern Jiangsu > central Jiangsu > northern Jiangsu”. The efficiency level of southern Jiangsu has always been in a leading position and has become the “main contributor” to the province’s efficiency. By contrast, cities with low efficiency are mainly located in central Jiangsu and northern Jiangsu. The efficiency fluctuations in these cities were more obvious. From a micro level, Zhenjiang had remained in the ranks of the “higher-efficiency area” for five years and was an “important contributor” to the province’s efficiency value. Suzhou, Changzhou, Yangzhou, and Nanjing have also stabilized at a relatively high efficiency level in the past five years. By contrast, Nantong and Lianyungang have always belonged to the “lower-efficiency zone”, and the efficiency values of Taizhou, Suqian, Yancheng, Huaian, and other cities were also relatively low. The efficiency of Xuzhou in 2018 and 2019 has fallen sharply. In general, different regions and cities had different investment scales, allocation methods, and management levels of medical and health resources due to differences in economic development and population distribution, which in turn causes spatial differences in the efficiency of medical and health services.

### 3.2. Results of the DEA-Malmquist Model

In terms of efficiency evaluation, the DEA-SE-SBM model can only measure the efficiency within a certain period, and cannot reflect the dynamic evolution of efficiency and the deep-seated reasons that affect efficiency changes. Therefore, this study used the Malmquist index model to decompose the total factor productivity of the medical and health services industry in Jiangsu Province, and further explore the deep-seated reasons that promote efficiency changes. Table 5 and Table 6 show the TFPCH and its decomposition in the time dimension and the geographical dimension, respectively.

It can be seen from Table 5 that the average TFPCH of medical and health services in Jiangsu Province from 2015 to 2019 was less than 1 (0.940), and the total factor productivity of medical and health services has fallen by 6% annually, indicating that the overall efficiency of medical and health services in the province showed a downward trend and characteristics. There is no obvious improvement. Among them, the TFPCH from 2018 to 2019 was the highest at 1.017, indicating that total factor productivity increased by 1.7%. The TFPCH from 2015 to 2016 and 2017 to 2018 were both less than 1 (0.857 and 0.890), indicating that total factor productivity decreased by 14.3% and 11%, respectively. From the perspective of decomposition indicators, the average values of EFFCH and TECHCH were both less than 1 (0.987 and 0.953). It shows that both the technical efficiency change index and the technology change index show a downward trend, which jointly restrict the increase in total factor productivity, and the latter has a greater impact than the former. The average values of PECH and SECH were 0.993 and 0.994, respectively, indicating that the pure technical efficiency and scale efficiency have decreased by 0.7% and 0.6% annually, respectively. The above analysis shows that focusing on the upgrading of medical equipment and the improvement of the skills and quality of health personnel were the main directions for improving the total factor productivity of medical and health services in Jiangsu Province. Improving the management level of medical and health services, strengthening resource input and utilization capabilities, and optimizing the scale of input and output are important driving forces for increasing the total factor productivity of medical and health services.

As can be seen from Table 6, the total factor productivity of medical and health services in the 13 cities was relatively large during the study period. Among them, Wuxi, Suzhou, and Zhenjiang achieved the total factor productivity growth. The largest increase was in Wuxi, with an increase of 10.1%. Investigating the deep reasons, the driving forces for the improvement of total factor productivity in the three cities included the following aspects: (1) The promoting role of technical progress, such as Suzhou and Zhenjiang. (2) The joint drive of technical progress and technical efficiency improvement, such as Wuxi.

By contrast, the remaining 10 cities such as Yancheng, Xuzhou, and Changzhou showed different degrees of decline. The largest decline was in Nantong, with a decrease of 21.8%. Further analysis found that the main reasons hindering the growth of total factor productivity in these cities included the following aspects: (1) The joint constraints of the decline in technical efficiency change index and technical change index. Among these cities, the decrease in Xuzhou, Yangzhou, and Taizhou was mainly due to the decline in pure technical efficiency. The decline, in Changzhou and Suqian, was due to the decline in scale efficiency. (2) The decline in technical change index, such as that in Lianyungang, Huaian, and Yancheng. Among these cities, the typical one was Lianyungang, which had a relatively low TECHCH of only 0.853. (3) The decline in technical efficiency change index. For example, in Nanjing, EFFCH was 0.969, indicating an average annual decline of 3.1% in technical efficiency. The decline in pure technical efficiency had a relatively effect on it.

### 3.3. Results of the Comprehensive Evaluation

**Figure 4:**
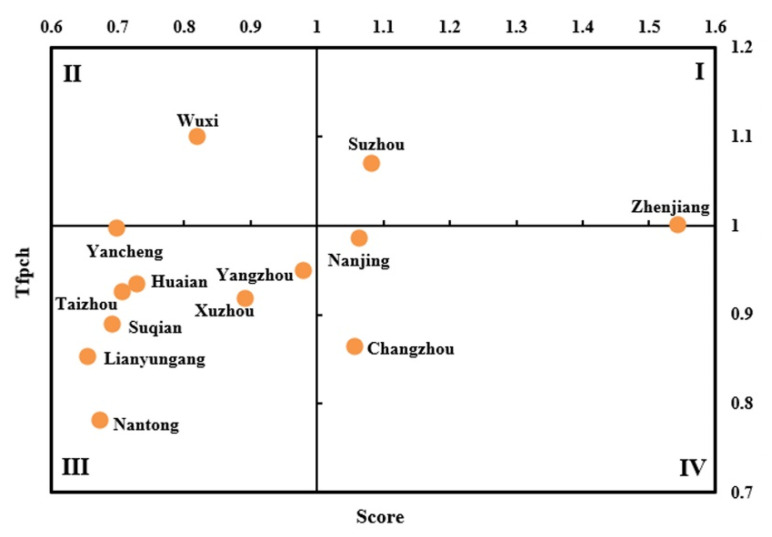
Classification of cities in Jiangsu Province based on the comprehensive measurement of efficiency from 2015 to 2019.

In order to comprehensively reveal the static and dynamic efficiency distributions of medical and health services in 13 cities during the study period, the efficiency value (Score) based on the DEA-SE-SBM model and the total factor productivity index (TFPCH) based on the DEA-Malmquist index model of each city from 2015 to 2019 were plotted as a quadrant distribution map (Figure 4). In the figure, cities with effective medical and health services efficiency and showing an upward trend are located in the first quadrant, including Suzhou and Zhenjiang. Cities with effective medical and health services efficiency but showing a downward trend are located in the fourth quadrant, mainly including Nanjing and Changzhou. The cities where the efficiency of medical and health services have not reached the effective state of DEA but showed a trend of improvement are in the second quadrant, including Wuxi. The cities in the third quadrant mainly include Xuzhou, Taizhou, Yancheng, and Yangzhou. The average efficiency and TFPCH of these cities are both less than 1. It is not difficult to find that there are more cities in the third quadrant, accounting for 61.54% (8/13). The efficiency of these cities is not as effective as DEA and is showing a downward trend, which should be taken seriously.

## 4. Results of Spatial Correlation Analysis of the Efficiency of Medical and Health Services

This study explored the spatial autocorrelation of the efficiency of 13 cities in Jiangsu Province so that we can further clarify the spatial correlation characteristics of the medical and health services efficiency and provide a scientific basis for the coordinated development of regional medical and health undertakings.

### 4.1. Results of Global Autocorrelation Analysis

Based on the medical and health service efficiency values calculated by the DEA-SE-SBM model, this study used Geoda 1.16 software to calculate the global Moran’s *I* of medical and health services efficiency of 13 cities in Jiangsu Province, from 2015 to 2019, and obtain their *Z* statistics test values and the significance level *P* value in order to judge the efficiency as a whole spatial relevance (Table 7).

It can be seen from Table 7 that, during the study period, the global Moran’s *I* of the medical and health services efficiency of each city in Jiangsu Province was always positive. Moreover, statistics in 2015 passed the significance test at the 10% level (*p* < 0.1), and the *Z* value was 1.8455, which was greater than the critical value of 1.69. Starting from 2016, the global Moran’s *I* passed the significance test at the 5% level (*p* < 0.05), and all other years passed the *Z* statistic test (*Z* > 1.96) except for 2016. The above analysis shows that, except for 2016, the medical and health services efficiency of various cities in Jiangsu Province are not randomly distributed in space, but have a significant positive correlation. The cities with similar medical and health services efficiency in the province are spatially clustered or dependent. In addition, the global Moran’s *I* from 2015 to 2019 showed a “barb-shaped” characteristic. The global Moran’s *I* in 2015, 2017, and 2018 showed an upward trend and characteristic year by year, and it declined slightly in 2019. On the whole, the spatial agglomeration effect of medical and health services efficiency continued to increase.

### 4.2. Results of Local Autocorrelation Analysis

In order to further measure the specific location of agglomeration or abnormal occurrence, local spatial autocorrelation analysis was carried out on the basis of global Moran’s *I* calculation. GeoDa 1.16 software and ArcGIS 10.2 software were used to obtain Moran scatter plot and LISA agglomeration map to reflect the statistical characteristics and specific locations of the local spatial agglomeration of the medical and health services efficiency.

**Figure 5:**
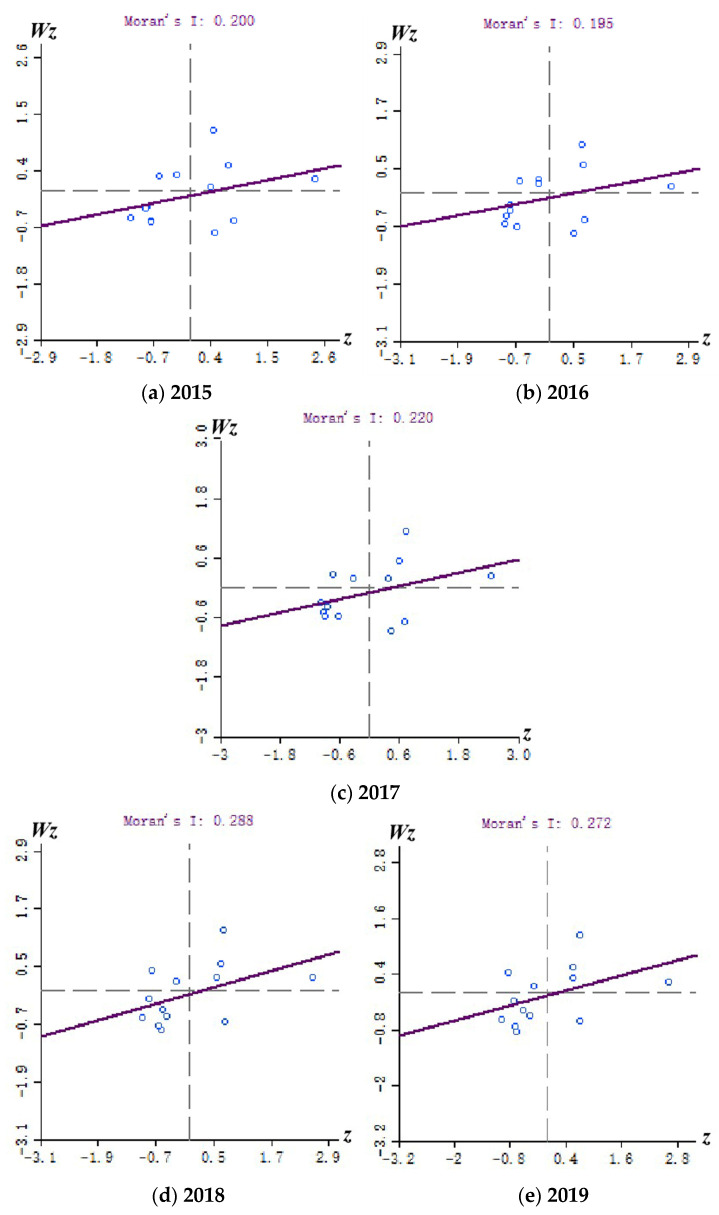
The Moran scatter plot of the local autocorrelation of medical and health services efficiency in Jiangsu Province from 2015 to 2019.

Combined with the Moran scatter plot (Figure 5), it can be found that most of the 13 cities in Jiangsu Province fell in the first and third quadrants (the agglomeration area and the depression area) in five years. The number of cities in the agglomeration area and the depression area in 2015 are 9, and the number rose to 10 in 2019. It further illustrates that the spatial agglomeration effect of medical and health services efficiency in Jiangsu Province transcended spatial heterogeneity. That is to say, the high-efficiency city tended to converge with high-efficiency city, and the low-efficiency city tended to converge with low-efficiency city. This phenomenon has a tendency to increase. However, it was worth noting that there were always more cities in L-L agglomeration areas than those in H-H agglomeration areas, which indicated that the degree of agglomeration of cities with lower-efficiency in Jiangsu Province was higher than those with higher-efficiency. It had a negative impact on the overall efficiency.

**Figure 6:**
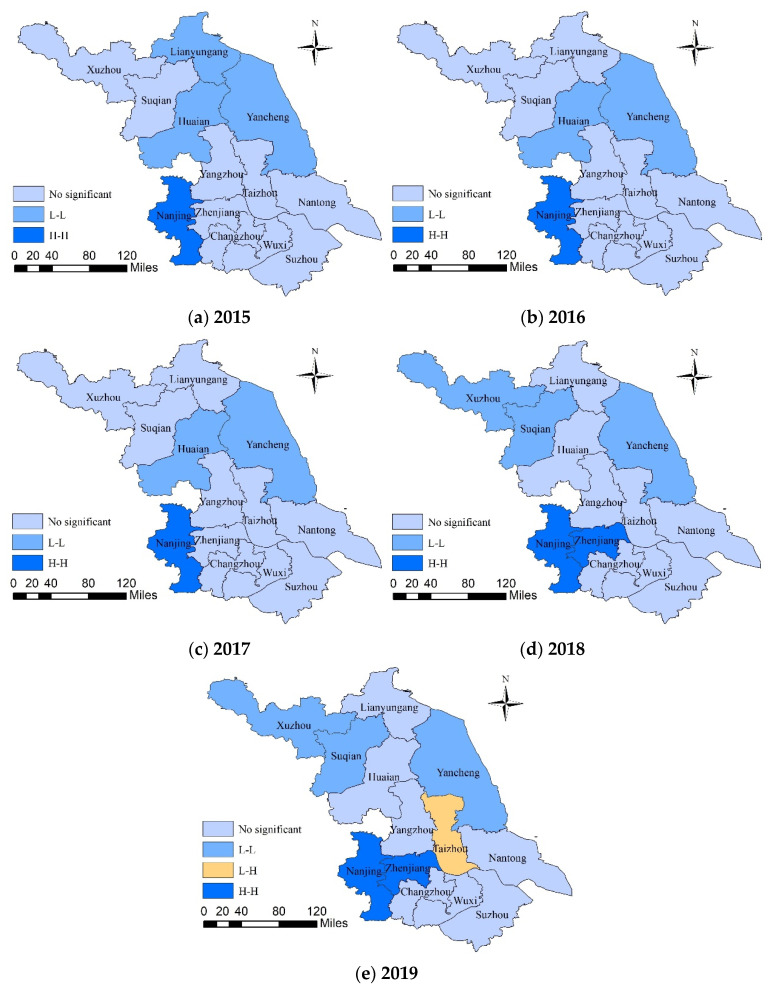
The LISA agglomeration map of the local autocorrelation of medical and health services efficiency in Jiangsu Province from 2015 to 2019.

Global Moran’s *I* acted as a regional overall measure indicator, and the scatter plot mainly performed descriptive statistics on the local spatial autocorrelation. Neither of them clearly described the type of efficiency level and the statistical significance of the local spatial correlation. Therefore, based on the global Moran’s *I* calculation and Moran scatter plot figure, the LISA agglomeration map of the medical and health services efficiency in Jiangsu Province from 2015 to 2019 was drawn to reflect the local spatial correlation characteristics of the efficiency (Figure 6).

It can be seen from the figure that, at the significance level of 5% and 10%, the spatial agglomeration characteristics of the medical and health services efficiency in Jiangsu Province from 2015 to 2019 were mainly L-L type and H-H type agglomeration, and they were distributed in contiguous areas. In 2015, a total of 4 cities passed the significance test. Among them, Nanjing was H-H type agglomeration and Yancheng, Huaian, and Lianyungang were L-L type agglomeration. In 2016 and 2017, 3 cities passed the significance test. Among them, Nanjing was H-H type agglomeration, and Huaian and Yancheng were L-L type agglomeration. In 2018, 5 cities passed the significance test. Among them, Nanjing and Zhenjiang were H-H type agglomerations, and Xuzhou, Suqian, and Yancheng were L-L type agglomeration. In 2019, 6 cities passed the significance test. Among them, the cities of H-H type and L-L type agglomeration were consistent with 2018, and Taizhou was newly added, which was L-H type agglomeration.

Further analysis found that the “hot spot areas” mainly include Nanjing and Zhenjiang in southern Jiangsu. Nanjing has always belonged to the H-H type agglomeration, indicating that the efficiency of medical and health services in Nanjing and neighboring cities has always been relatively high. Zhenjiang also joined the H-H type agglomeration in 2018. The “cold spot areas” is mainly composed of cities in northern Jiangsu. Yancheng has always been L-L type agglomeration, indicating that the medical and health services of this city and its neighboring cities have been low. Huaian dropped out of L-L type agglomeration in 2018. Xuzhou and Suqian entered the ranks of L-L type agglomeration in 2018. The economic development and health management in northern Jiangsu were relatively backward; moreover, areas with high-efficiency, such as Nanjing and Zhenjiang, had limited radiation and driving effects in northern Jiangsu. Therefore, most cities in northern Jiangsu showed a positive spatial correlation of low efficiency. It is worth noting that Taizhou changed from L-L type agglomeration to L-H type agglomeration in 2019. The main reason is that, compared with neighboring cities, the city’s efficiency value in 2018 was low (0.664), and the efficiency further reduced in 2019 (0.632).

## 5. Discussion 

From 2015 to 2019, the average medical and health services efficiency in Jiangsu Province showed the characteristics of “decline-rise-decline”; they were all less than 1, ranging from 0.860 to 0.930. The number of cities with effective efficiency in each year did not exceed half. The efficiency of medical and health services varied significantly between regions. The spatial distribution pattern of efficiency presented a trend of “southern Jiangsu > central Jiangsu > northern Jiangsu”. This conclusion is similar to the research results of Ben et al. [21]. Nanjing, Changzhou, Suzhou, and other cities in southern Jiangsu had relatively high efficiency levels over the years. Nantong, Huaian, Lianyungang, and other cities in central and northern Jiangsu had lower efficiency levels. In general, the efficiency of medical and health services in the 13 cities in Jiangsu Province had not yet reached the optimal level, and there was room for improvement. Based on this, in the future development of medical and health services, the government must focus on optimizing the allocation of medical and health resources and improving the efficiency of medical and health services. For different cities, the government should adjust measures to local conditions and implement different plans based on clarifying the allocation level of its own medical and health resource and the residents’ preference for medical treatment and health needs, so as to gradually achieve a balanced allocation of high-quality medical and health resources and further expand effective medical and health services to promote efficiency.

During the study period, the average TEPCH of medical and health services in 13 cities in Jiangsu Province was 0.940, experiencing an average annual decrease of 6%. The decline was mainly due to the joint constraints of reduced technical efficiency and technical changes, and the effect of the latter was greater than the former. This is consistent with the conclusion of the study of Hunan by Zhong et al. [48]. In order to solve this problem, some cities in the province should increase the introduction of high-tech medical equipment and high-level health personnel in order to improve the level of medical and health technology, thereby increasing the total factor productivity. On the one hand, some cities should pay attention to the introduction of high-tech equipment and establish a supervision mechanism to promote the rational use of equipment and reduce unnecessary inspections and procedures for patients. On the other hand, some cities should strive to make up for shortcomings in medical and health informatization, apply fast-developing information technology to the construction of an efficient medical and health service system, actively explore “Internet + medical”, and make full use of the Internet of Things, big data, and cloud computing and other technologies to promote the quality and efficiency of urban medical and health services. In addition, we should focus on continuously optimizing the soft power of medical and health and strengthen the introduction and training of talents in the medical and health field.

The global Moran’s *I* of the medical and health services efficiency of all cities in Jiangsu Province were all positive from 2015 to 2019. Except for 2016, all other years passed the significance test and the *Z* statistic test, indicating that the medical and health services efficiency of Jiangsu Province was not randomly distributed in space, but had a certain spatial relevance. According to the first law of geography, proximity makes things more relevant [49]. The medical and health services efficiency of each city was not only related to its own medical and health development, but also affected by the efficiency of the corresponding surrounding cities. That is to say, there was a “positive feedback” effect in space. This is consistent with the conclusion obtained by Liu et al. in their spatial analysis of the efficiency of rural basic public health services in China [50]. In addition, the spatial agglomeration effect of medical and health services efficiency continued to increase from 2015 to 2018, and there was only a slight decrease in 2019. Therefore, Jiangsu Province should further strengthen the cooperation and exchanges between cities in the distribution and utilization of medical and health resources and the innovation of medical and health service models under the premise of fully considering the differences in geographical space, population distribution, and economic development of various cities. Meanwhile, each city should not only pay attention to the improvement of its own medical and health services efficiency, but also pay attention to the operation and change trend of the medical and health system of neighboring cities to avoid its negative spatial impact.

The LISA agglomeration map verifies that at the significance level of 5% and 10%, the spatial agglomeration characteristics of the medical and health services efficiency of Jiangsu Province from 2015 to 2019 were mainly L-L type and H-H type agglomeration, and it presented the pattern of contiguous area distribution. The “hot spot areas” mainly included Nanjing and Zhenjiang, and the “cold spot areas” mainly included Lianyungang, Huaian, Yancheng, and other cities. L-H type agglomeration only appeared in 2019. It is Taizhou City, which is related to the further reduction in its efficiency value. There was no H-L type agglomeration. In this regard, cities in the “hot spot areas” should develop their own medical and health advantages; at the same time, they should emphasize the leading role of the surrounding radiation and practice the regional assistance model. Specifically, the excellent medical system internal management methods and technology utilization experience that have been practiced should be shared with low-efficiency cities. Moreover, they should gradually share high-quality medical and health personnel, technology, information, and other resources across regions to promote the efficiency of surrounding cities. The cities in the “cold spot areas” can strengthen the internal management level of the medical and health system by imitating the excellent practices of high-efficiency cities. Then, they can continuously optimize the medical and health personnel and technical capabilities of the district through regional joint adjustments to promote continuous improvement of medical and health services efficiency. Finally, the provincial government’s policies should be tilted toward low-efficiency areas; at the same time, relevant evaluation and review mechanisms should be established to promote their implementation of the medical and health service system planning.

## 6. Conclusions

This study took Jiangsu Province as an example and selected the panel data of the province’s medical and health input and output from 2015 to 2019. First, based on the DEA-SE-SBM model, a static evaluation of the medical and health services efficiency of 13 cities in the province was carried out. At the same time, the DEA-Malmquist model was used to explore the dynamic evolution of efficiency and the deep-seated reasons affecting its changes. Further, the ESDA method was used to explore the spatial autocorrelation of the medical and health services efficiency. The main conclusions are as follows.

Firstly, During the study period, the efficiency of medical and health services in 13 prefecture-level cities in Jiangsu Province was relatively good, but there were regional differences in efficiency, and there was room for improvement.

Secondly, the total factor productivity of medical and health services in Jiangsu Province showed a downward trend and characteristic from 2015 to 2019; technical change had a greater impact on it.

Finally, the results of global spatial autocorrelation analysis show that there was a certain spatial correlation in the efficiency of medical and health services in 13 cities in Jiangsu Province. Through local spatial autocorrelation analysis, it can be found that the “hot spot areas” mainly include some cities in southern Jiangsu, the “cold spot areas” mainly include cities in northern Jiangsu, and the number of the latter is greater than the former.

This paper mainly used the data of Jiangsu Province from 2015 to 2019 to conduct an analysis of comprehensive measurement and spatial correlation of efficiency between cities. The internal and external factors that promote the efficiency of this spatial pattern, such as urbanization and population mobility, were less considered. It needs to be further studied and discussed in future research to provide a deeper explanation of the spatial differentiation characteristics of the efficiency of Chinese medical and health services.

## Figures and Tables

**Table 1:** Distribution of prefecture-level cities in the three regions of Jiangsu Province.

Region	Prefecture-Level City
southern Jiangsu	Zhenjiang, Nanjing, Changzhou, Suzhou, Wuxi
central Jiangsu	Yangzhou, Taizhou, Nantong
northern Jiangsu	Lianyungang, Suqian, Huaian, Yancheng, Xuzhou

**Table 2:** The index system of health service efficiency evaluation.

Items	Specific Indicators	Explanation of Indicators	Significance of Indicators	References
Input	X_1_: Number of health technicians (person)	Health technicians mainly include registered nurses, licensed (assistant) physicians, pharmacists, health supervisors, technicians, and other health technicians.	Reflecting the investment scale of medical and health human resources in each year in the study area.	[5,9,21,48]
	X_2_: Number of Health Institutions (unit)	Health Institutions mainly include hospitals, professional public health institutions, primary medical and health institutions, and other institutions.	Reflecting the input of medical and health material resources in each year in the study area.	[20,24,29,30,47]
	X_3_: Number of Beds (unit)	The number of beds is the sum of the number of beds in various medical and health institutions each year.		[9,20,27,28,46,47]
Output	Y_1_: Number of Outpatients and Emergency Visits (10,000 person)	Outpatient and emergency visits are the sum of outpatient visits and emergency visits.	Reflecting the supply of medical and health services of the research subjects in that year.	[9,23,28,48]
	Y_2_: Utilization Rate of Beds (%)	The utilization rate of beds indicates the utilization of beds in all medical and health institutions in each region.		[5,24,27]

**Table 3:** Medical and health input-output of 13 cities in Jiangsu Province.

Region	Cities	2015					2019				
		X_1_	X_2_	X_3_	Y_1_	Y_2_	X_1_	X_2_	X_3_	Y_1_	Y_2_
southern Jiangsu	Nanjing	65,139	2337	46,643	7010.00	89.84	93,856	3242	59,046	9070.70	91.17
	Wuxi	44,707	2243	37,366	4856.00	86.82	59,303	2770	50,478	5786.16	79.42
	Changzhou	29,616	1196	24,263	2804.00	98.06	37,086	1458	28,322	3223.91	90.53
	Suzhou	68,179	3121	59,304	8968.00	88.34	91,047	3720	71,657	10,085.47	86.52
	Zhenjiang	18,985	943	14,637	2326.00	77.90	21,691	1013	15,844	2484.23	84.99
central Jiangsu	Nantong	41,067	3147	36,031	3908.00	93.53	50,329	3357	46,375	4253.57	87.99
	Yangzhou	24,326	1780	20,121	2592.00	90.62	29,406	1890	24,994	2588.01	92.00
	Taizhou	24,215	1953	21,838	2405.00	87.32	31,673	2118	29,885	2508.03	86.45
northern Jiangsu	Xuzhou	51,567	4601	47,949	5880.00	99.74	70,767	4594	60,988	6245.99	87.19
	Lianyungang	23,056	2708	19,035	2729.00	75.90	31,117	2740	28,101	2859.41	75.85
	Huaian	30,475	2228	25,966	2776.00	91.01	35,963	2200	30,376	2731.71	88.64
	Yancheng	39,494	3242	37,169	3986.00	80.67	44,358	3270	40,301	4767.89	81.99
	Suqian	26,179	2426	23,290	2929.00	77.74	36,749	2424	29,548	3344.65	78.21

Note: The study takes 2015 as the base period to analyze the medical and health services efficiency of 13 cities in Jiangsu Province from 2015 to 2019. Therefore, only the medical and health input-output of 2015 and 2019 are displayed and compared here.

**Table 4:** Medical and health services efficiency value of Jiangsu Province from 2015 to 2019.

Cities	2015	2016	2017	2018	2019	Mean
Nanjing	1.028	1.070	1.089	1.063	1.063	1.063
Wuxi	0.859	0.837	0.820	0.799	0.786	0.820
Suzhou	1.123	1.084	1.079	1.064	1.064	1.083
Changzhou	1.096	1.080	1.050	1.041	1.018	1.057
Zhenjiang	1.495	1.547	1.521	1.555	1.600	1.544
Taizhou	0.780	0.740	0.717	0.664	0.632	0.706
Nantong	0.724	0.685	0.650	0.647	0.663	0.674
Yangzhou	1.016	0.838	1.000	1.022	1.024	0.980
Lianyungang	0.741	0.665	0.667	0.607	0.593	0.655
Xuzhou	1.034	1.024	1.012	0.714	0.678	0.893
Huaian	0.745	0.726	0.739	0.719	0.718	0.729
Yancheng	0.649	0.661	0.675	0.746	0.759	0.698
Suqian	0.718	0.690	0.687	0.698	0.670	0.692
Mean	0.924	0.896	0.900	0.872	0.867	0.892

**Table 5:** Malmquist index of medical and health services in Jiangsu Province and its decomposition during 2015–2019 (time dimension).

Year	EFFCH	TECHCH	PECH	SECH	TFPCH
2015—2016	1.009	0.849	1.029	0.981	0.857
2016—2017	1.004	1.004	0.996	1.009	1.009
2017—2018	0.995	0.894	0.984	1.011	0.890
2018—2019	0.941	1.082	0.963	0.977	1.017
Mean	0.987	0.953	0.993	0.994	0.940

**Table 6:** Malmquist index of medical and health services in Jiangsu Province and its decomposition during 2015–2019 (geographical dimension).

Cities	EFFCH	TECHCH	PECH	SECH	TFPCH
Wuxi	1.032	1.067	1.027	1.005	1.101
Suzhou	1.000	1.070	1.000	1.000	1.070
Zhenjiang	1.000	1.001	1.000	1.000	1.001
Yancheng	1.000	0.997	1.000	1.000	0.997
Nanjing	0.969	1.018	0.974	0.995	0.986
Yangzhou	0.991	0.959	0.991	1.000	0.950
Huaian	1.004	0.932	1.010	0.993	0.935
Taizhou	0.986	0.939	0.960	1.028	0.926
Xuzhou	0.973	0.944	0.968	1.005	0.918
Suqian	0.953	0.934	1.000	0.953	0.890
Changzhou	0.993	0.871	1.039	0.955	0.864
Lianyungang	1.000	0.853	1.000	1.000	0.853
Nantong	0.934	0.837	0.940	0.994	0.782
Mean	0.987	0.953	0.993	0.994	0.940

**Table 7:** The global Moran’s *I* value of the efficiency of medical and health services in Jiangsu Province from 2015 to 2019.

Year	2015	2016	2017	2018	2019
Moran’s *I*	0.200	0.195	0.220	0.288	0.272
*Z*	1.8455	1.8902	1.9777	2.4557	2.3843
*P*	0.051	0.046	0.039	0.017	0.021

## Data Availability

The data presented in this study are available on request from the corresponding author. The data are not publicly available due to legal and privacy issues.

## Abbreviations

DEA	Data Envelopment Analysis;
SFA	Stochastic Frontier Analysis;
BCC	Banker, Charnes, and Cooper model;
CCR	Charnes, Cooper, and Rhodes model;
SBM	slack-based measure model;
SE-SBM	super efficiency slack-based measure model;
ESDA	Exploratory Spatial Data Analysis;
DMUs	Decision-making units;
TFPCH	Total Factor Productivity Change index;
EFFCH	Technical Efficiency Change index;
TECHCH	Technical Change index;
VRS	variable returns to scale;
PECH	Pure Efficiency Change index;
SECH	Scale Efficiency Change index;
LISA	Local Indicator of Spatial Association;
